# ST Elevation Infarction after Heart Transplantation Induced by Coronary Spasms and Mural Thrombus Detected by Optical Coherence Tomography

**DOI:** 10.1155/2016/1863869

**Published:** 2016-11-17

**Authors:** Tor Skibsted Clemmensen, Niels Ramsing Holm, Hans Eiskjær, Steen Hvitfeldt Poulsen, Michael Maeng, Christian Juhl Terkelsen, Evald Høj Christiansen

**Affiliations:** Department of Cardiology, Aarhus University Hospital, Aarhus, Denmark

## Abstract

The case illustrates the possible link between coronary spasms, intraluminal thrombus formation, and widespread organized and layered thrombi in HTx patients. Furthermore, the case underlines the clinical value of OCT as a novel method for high-resolution vessel imaging in heart-transplanted (HTx) patients with coronary spasms and suspected coronary artery disease. Coronary spasms and sudden death are frequent complications after HTx. The underlying mechanisms leading to these complications are unknown. The present case displays the clinical course of a 19-year-old HTx patient who was hospitalized due to acute myocardial infarction induced by severe coronary spasms. The patients remained unstable on conservative therapy. Therefore, an optical coherence tomography (OCT) was performed and revealed massive, organized thrombi in the left main coronary artery, the circumflex coronary artery, and the left anterior descending coronary artery. The patient was stabilized after percutaneous coronary intervention. As a mural thrombus often goes undetected by coronary angiography, OCT may prove benefit in HTx patients with myocardial infarction or suspected coronary spasms.

## 1. Introduction

Cardiac allograft vasculopathy (CAV) remains the most frequent cause of long-term heart-related mortality after heart transplantation (HTx) [1]. Furthermore, coronary spasms and sudden death can occur at any time-point after HTx. The underlying mechanisms to these complications remain unknown. In order to determine CAV severity and progression it is recommended to perform annually or biannually coronary angiography [2]. However, conventional angiography often misses and underestimates the burden of CAV. Hence, in approximately 50% of angiographic normal vessels intravascular ultrasound (IVUS) diagnoses CAV [3, 4]. IVUS is therefore widely used in CAV surveillance [2]. In recent years, optical coherence tomography (OCT) has emerged as a sophisticated high-resolution intravascular imaging modality. Therefore, OCT may provide important, so far undetectable* in vivo* insights into the pathogenesis of CAV and coronary spasms.

## 2. Case Presentation

A 19-year-old HTx female patient with 12 hours of on/off chest pain and syncope was hospitalized in September 2015 due to suspected ST-segment elevation myocardial infarction. She was transplanted without complications in May 2010 due to terminal arrhythmogenic right ventricular cardiomyopathy. The patient had no rejections during 2 years of routine biopsy surveillance, and coronary arteries appeared normal during the first 4 years after HTx assessed by annual angiography. The patient received immunosuppression with Tacrolimus and Mycophenolate and received no antiplatelet therapy.

**Figure 1:**
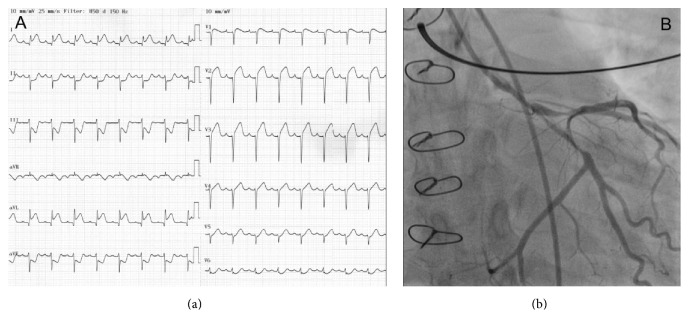
(a) Electrocardiogram at the time of admission showing global ischemia. (b) Acute coronary angiography showing severe coronary spasms in the left main, circumflex, and left anterior descending coronary artery.

**Figure 2:**
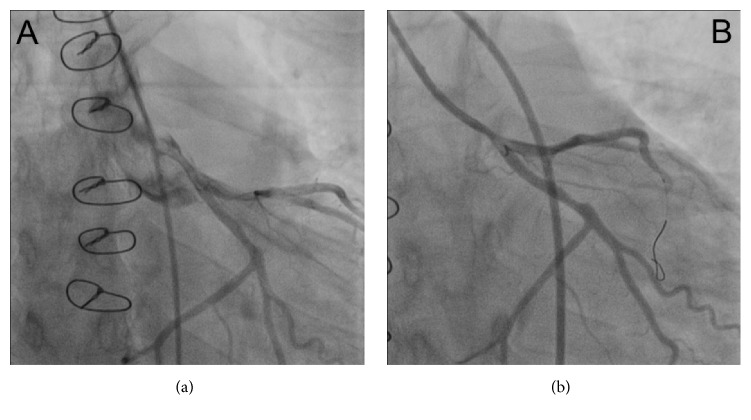
(a) Coronary angiography from the day after admission with only a possible spasm in the left main coronary artery (LMCA) and no spasms in the left anterior descending or circumflex (CX) coronary artery. (b) Third coronary angiography performed 6 days after admission showing LMCA and ostial CX stenosis.

A prehospital electrocardiogram (ECG) showed global ischemia. An acute coronary angiography revealed severe coronary spasms in the left main coronary artery (LMCA), circumflex (Cx), and left anterior descending coronary artery (LAD) (Figure 1, Supplementary Movie 1) (see Supplementary Material available online at http://dx.doi.org/10.1155/2016/1863869). However, the ECG ST-segment deviations normalized during initial treatment with intravenous nitroglycerine and low-molecular-weight heparin. No interventional treatment was therefore performed. Blood samples confirmed a large myocardial infarction with troponin-T of 9649 ng/L and CKMB of >600 *μ*g/L. Echocardiography revealed a reduced left ventricular systolic function with an ejection fraction of 35% and a global longitudinal strain −10.9% (Supplementary Movie 2). Endomyocardial biopsies showed no signs of acute cellular or humoral rejection, and Luminex analysis of donor-specific antibodies was negative. A new coronary angiography on day two revealed only a possible spasm or stenosis in the distal LMCA (Figure 2(a), Supplementary Movie 3). A class III calcium antagonist was subscribed along with protracted-action nitroglycerine.

**Figure 3:**
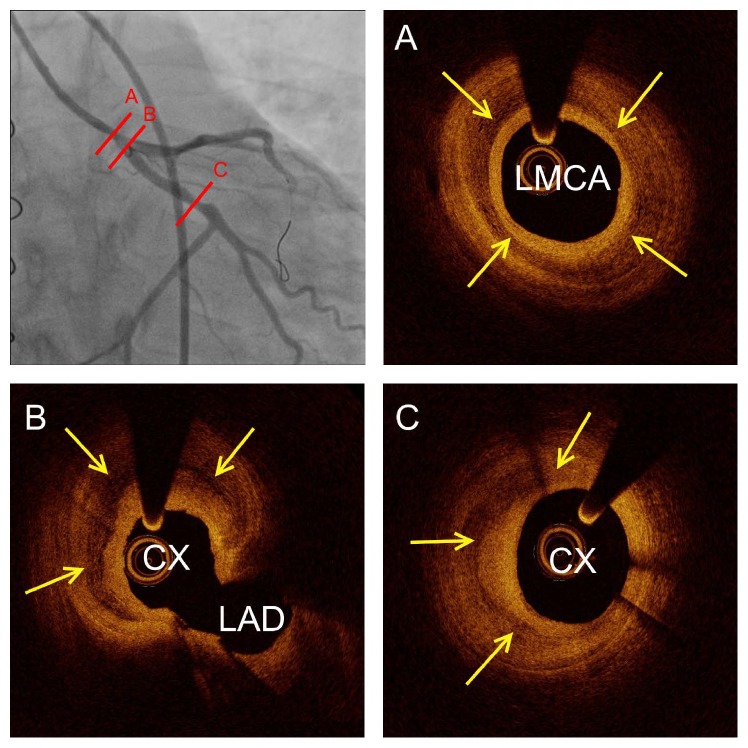
Intravascular optical coherence tomography at the angiographic lesion sites. (A) Circumflex layered massive organized thrombus in the left main coronary artery (LMCA) covering the ostium of the circumflex (CX) and left anterior descending coronary artery (LAD) (B). Furthermore, crescent-shaped layered organized thrombus was seen in the distal part of the circumflex (C). Yellow arrows show layered thrombosis within the intima vessel layer.

As the patient continued to present with intermittent chest pain and dynamic ST-segment deviations, a third coronary angiography (Figure 2(b), Supplementary Movie 4) was performed on day 6 including optical coherence tomography (OCT). The angiography showed LMCA and ostial Cx stenosis, and the OCT revealed a massive, organized thrombus in the LMCA involving the ostium of the Cx. Widespread, organized, and partly organized, layered thrombi were found in the LMCA, Cx, and LAD (Figure 3, Supplementary Movie 5). Treatment of the LMCA and Cx bifurcation was performed with the culotte two-stent technique using OCT guidance, thereby obtaining a satisfying result (Supplementary Movie 6 and Supplementary Movie 7). The patient was without symptoms the following days and was discharged 7 days later in habitual conditions.

## 3. Discussion

The case illustrates the possible link between coronary spasms, intraluminal thrombus formation, and widespread organized and layered thrombi in HTx patients. Furthermore, the case underlines the clinical value of OCT as a novel method for high-resolution vessel imaging in HTx patients with coronary spasms and suspected coronary artery disease. It is possible that OCT use in the early phase of the present case after nitroglycerine loading and resolution of spasms would have revealed the thrombus formation in the LMCA, and a more aggressive antithrombotic strategy or percutaneous intervention could have been chosen. As a mural thrombus often goes undetected by coronary angiography, intravascular imaging may be recommended in HTx patients with myocardial infarction or suspected coronary spasms. The two main intravascular imaging modalities are OCT and IVUS. OCT has 10 times greater spatial resolution than IVUS. Therefore, it enables a more detailed, so far undetectable* in vivo* vessel wall microstructure evaluation in HTx patients. In the present case, IVUS would have shown increased intima thickness, but the underlying cause of intima hypertrophy would be unclear. The main disadvantage of OCT to IVUS is the contrast use. The average contrast use for OCT is 20–30 contrast per vessel. This additional contrast use must be carefully considered in HTx patients with advanced renal dysfunction (creatinine > 200 *μ*mol/L).

Coronary spasms and sudden death are well known complications after HTx. Treatment of coronary spasms consists of medical stabilization as the location of the spasm is likely to vary with time. HTx patients develop cardiac allograft vasculopathy (CAV) at high rates [1]. Traditionally, CAV is described as concentric intimal thickening mediated by inflammation and proliferation plus migration of smooth muscle cells from the media layer [5]. However, recent reports based on virtual histology intravascular ultrasound [6] and OCT [7] have revealed a high prevalence of layered complex plaques, which may represent organized thrombi and could be a significant component of the accelerated intimal thickening in HTx patients. The present case is the first report to indicate a potential link between coronary spasms and complex layered thrombotic plaques in HTx patients. The underlying pathophysiological mechanism of layered complex plaque formation could be directly linked to spasms-induced flow disturbances. However, the clinical significance and mechanisms of these findings warrant further evaluation.

## Supplementary Material

Movie Legends:
Movie I: Acute coronary angiography with severe spasms in the LMCA, Cx, and LAD.
Movie II: Echocardiography showing reduced longitudinal strain and wall motion in the LAD territory.
Movie III: Second coronary angiography with no spasms in the Cx and LAD, but suspected spasms in the LMCA.
Movie IV: Third coronary angiography and percutaneous intervention of the LMCA and Cx bifurcation performed by the culotte two-stent technique.
Movie V: OCT images of the LMCA and LAD prior to percutaneous intervention.
Movie VI: OCT images of the Cx and LMCA after percutaneous intervention.
Movie VII: OCT images of the LAD and LMCA after percutaneous intervention.

## Additional Points

*Limitations*. We do not have histological validation of our OCT findings. Therefore, future histological studies should address if layered complex plaques are associated with organized thrombosis.
